# Gold Electrodes Modified with Calix[4]arene for Electrochemical Determination of Dopamine in the Presence of Selected Neurotransmitters

**DOI:** 10.3390/s17061368

**Published:** 2017-06-13

**Authors:** Katarzyna Kurzątkowska, Serkan Sayin, Mustafa Yilmaz, Hanna Radecka, Jerzy Radecki

**Affiliations:** 1 Department of Biosensors, Institute of Animal Reproduction and Food Research Polish Academy of Science, Tuwima 10 Street, Olsztyn 10-748, Poland; k.kurzatkowska@pan.olsztyn.pl (K.K.); h.radecka@pan.olsztyn.pl (H.R.); 2 Department of Environmental Engineering, Faculty of Engineering, Giresun University, Giresun-28200, Turkey; serkan.sayin@giresun.edu.tr; 3 Department of Chemistry, Selcuk University, Konya 42100, Turkey; myilmaz24@yahoo.com

**Keywords:** calix[4]arene, neurotransmitters, dopamine, norepinephrine, epinephrine, molecular recognition, voltammetric sensors

## Abstract

Here, we present an electrochemical sensor based on gold electrodes modified with calix[4]arene functionalized with carboxypiperidino groups at the upper rim. It has been demonstrated that these groups are involved in a complex formation with dopamine (DA) on the surface of gold electrodes. The supramolecular complex calix[4]arene–DA created on the gold electrode surface has been characterized electrochemically and the measuring conditions have been optimized. The presented sensor displayed a detection limit in the pM range. The DA determination was performed successfully in the presence of ascorbic acid, uric acid and selected neurotransmitters.

## 1. Introduction

Dopamine is a very important biomolecule belonging to the catecholamine neurotransmitters family. A basal level of dopamine (DA) is 1 nM and 10 nM in human serum and in the brain, respectively [1]. The deviations from the physiological concentrations of DA in the central nervous system could be a reason for many such medical conditions as depression and schizophrenia, as well as such neurodegenerative diseases as Parkinson’s, Alzheimer’s and Huntington’s [2,3]. For example, loss of dopamine in the brain of a Parkinson’s patient is in the range from −9 to −97%; furthermore, the decrease of DA is dependent on the different brain areas [4]. Therefore, the control of DA concentration in physiological fluids is one of the most important tasks in medical diagnosis.

In the analytical methods currently available for DA determination in physiological fluids, the separation step is involved in chromatography, enzyme immunoassay and mass spectrometry [5,6,7]. All of these methods require expensive instruments and highly educated staff. This is a significant obstacle in the broad application of these methods in the majority of medical diagnostic laboratories. 

The electroactivity of the catecholamine neurotransmitters family is often used as the base for their direct determination. Electrochemical methods (amperometry, differential pulse voltammetry, cyclic voltammetry) attract scientific interest because of their simplicity and sensitivity. The devices used in such types of analysis are relatively cheap and easily miniaturized [8]. The weak point of direct electrochemical methods is the overlapping of the electrochemical signals generated by redox active interfering substances, such as ascorbic and uric acids, which are present in serum and could also have an influence on neurotransmitters determination [9,10].

The modification of electrode surfaces with enzymes, such as with laccase and/or nanomaterials, could overcome these problems [11,12,13]. However, selectivity of the enzymatic sensors destined for catecholamine determinations is limited because of high enzymes reactivity towards phenolic groups. Therefore, distinguishing the presence of dopamine over the presence of other catecholamine neurotransmitters while using enzyme based sensors, is very limited [14]. More successful selectivity for the determination of dopamine in the presence of other neurotransmitters has been achieved by using sensors incorporated in the specific aptamer as a recognition element. The sensitivity of these sensors is, nonetheless, relatively low [1,15,16].

Here, we propose calix[4]arene as the sensing element in the electrochemical sensors destined for dopamine determination. The calixarenes belong to cavity-shaped cyclic compounds which are broadly applied in a supramolecular recognition process as host molecules [17,18]. A number of studies relating to the calixarenes and their derivatives have shown that they are very sensitive, and selective recognition elements in electrochemical sensors are destined for metal ions determination [19,20]. The calixarenes-based sensors are also suitable for the determination of small biomolecules [18,21,22,23].

In our previous paper, we introduced the electrochemical sensor incorporating calix[4]arene as a sensing element for dopamine determination [21]. This sensor generates an analytical signal according to an ion-channel mechanism [24,25,26]. A linear dynamic range in the dopamine concentration range from 1.0 × 10^−11^ to 1.0 × 10^−6^ M was achieved [21]. The weak point of this sensor, from a diagnostic point of view, is the need to add the redox marker to the sample solution.

In the present work, we describe the sensor based on gold electrode modified with calix[4]arene derivatives functionalized with mercaptoalkyl groups at the lower rim, and carboxypiperidino groups on the upper rim. In this sensor our strategy for analytical signal generation and improving selectivity is based on the formation of an electroactive calix[4]arene–DA supramolecular complex located at the surface of the gold electrode. Its electroactivity originates from dopamine. Applying this strategy, we eliminate the need to add the redox marker to the sample solution. The presented sensor is designed for the determination of dopamine in the presence of a significant concentration of another neurotransmitter, as well as ascorbic and uric acid.

## 2. Materials and Methods 

### 2.1. Reagents and Materials

The receptor 5,17-bis[*N*-(4-carboxypiperidino)methyl]-25,27-bis(3-mercapto-propoxy)-26, 28-dihydroxycalix[4]arene (HSCX-COOH) was synthesized by a previously reported procedure [20]. The chemicals 2-mercaptoethanol (MET), uric acid (UA), ascorbic acid (AA), dopamine hydrochloride (DA), norepinephrine hydrochloride (NEP), epinephrine hydrochloride (EP), citric acid, potassium chloride, sodium chloride, sodium hydrogen phosphate, potassium dihydrogen phosphate and dichloromethane, were purchased from Sigma–Aldrich (Poznań, Poland). Alumina slurry of 0.3 and 0.05 μm was purchased from Buehler (Lake Bluff, IL, USA). Potassium hydroxide, sulphuric acid and methanol were obtained from Advantor (Gliwice, Poland). 

All aqueous solutions were prepared with deionized and charcoal-treated water (resistivity of 18.2 MΩ cm^−1^) purified with a Milli-Q reagent grade water system (Millipore, Bedford, MA, USA). All solutions were deoxygenated by purging them with nitrogen (ultra-pure 6.0, Air Products, Olsztyn, Poland) for 15 min.

### 2.2. Electrode Preparation

Gold disk electrodes with a radius of 2 mm (Bioanalytical Systems (BAS), West Lafayette, IN, USA) were used for the modification. These electrodes, after being washed with methanol and Milli-Q water, were polished in alumina slurries, with particles in the size of 0.3 and 0.05 μm on microcloth polishing pads for 5 min each. Afterwards, they were carefully washed with Milli-Q water. Then, electrochemical cleaning was performed by cyclic voltammetry (CV). The first step involved a 0.5 M potassium hydroxide solution, wherein the potential was swept with a scan rate of 100 mV s^−1^ between −400 and −1200 mV (versus Ag/AgCl reference electrode). The gold electrodes were first submitted to 3 cycles, then to 50 additional cycles, and finally to 10 more cycles. All of these cycles were performed under the same conditions. Next, the electrodes were cleaned in a 0.5 M sulphuric acid solution. The potential was swept with a scan rate of 100 mV s^−1^ between −300 and +1500 mV (versus Ag/AgCl reference electrode). Initially, they were submitted to 3 cycles, then to 10 cycles, and finally to 3 more cycles, performed under the same conditions. After finishing the electrochemical cleaning, each electrode was rinsed with Milli-Q water and stored in water (for several minutes, until the next step) to avoid contamination from air. 

Directly after cleaning, the electrodes were dipped into 0.01 mM solutions of HSCX-COOH in a CH_2_Cl_2_/MeOH mixture (1/1, *v*/*v*) at room temperature for 3 h. Next, they were exposed to a 1.0 mM solution of MET in CH_2_Cl_2_ for 0.5 h at room temperature. 

The modification solutions were put into tubes (with no flat bottom). After the electrodes were dipped, the tubes were sealed with Teflon tape and parafilm to prevent any solvent evaporation. After the modification, electrodes were washed with CH_2_Cl_2_, MeOH and finally Milli-Q water. All modified gold electrodes were stored at 4 °C in a buffer solution (phosphate-buffered saline [PBS] pH 7.4 or 0.2 M McIlvain buffer pH 7.0) until use.

### 2.3. Electrochemical Measurements

All electrochemical measurements were performed with a potentiostat–galvanostat AutoLab (Eco Chemie, Utrecht, The Netherlands) with a three-electrode configuration. Gold electrode was used as a working electrode. Potentials were measured versus the Ag/AgCl electrode, and a platinum wire was used as an auxiliary electrode.

The study of the pH influence on redox activity of DA was performed with Osteryoung square-wave voltammetry (OSWV) in a potential range from −100 mV to +600 mV, and with a step potential of 1 mV, a square-wave frequency of 25 Hz, and an amplitude of 50 mV in the buffer solution. The determination of dopamine in the presence of the pure buffer, and the buffer with interferences, was studied using OSWV with a potential from −100 mV to +600 mV, and with a step potential of 0.5 mV, a square-wave frequency of 10 Hz, and an amplitude of 25 mV in the buffer solution. In CV, potentials were cycled from −600 to +600 mV with a scan rate 100 mV s^−1^. As a supporting electrolyte, a PBS buffer pH 7.4 (137 mM NaCl, 2.7 mM KCl, 10 mM KH_2_PO_4_, 1.8 mM Na_2_HPO_4_ solution) and 0.2 M McIlvain buffer pH 7.0 or 2.0 (0.4 M Na_2_HPO_4_ and 0.5 M citric acid, mixed in an appropriate ratio). 

All measurements were carried out in the presence of an electrolyte purged with nitrogen for 15 min. A gentle nitrogen flow was applied over the sample solution during all measurements.

## 3. Results and Discussion 

### 3.1. Electrochemical Characterization of Gold Electrode Modified with HS-CXCOOH in the Presence of Dopamine

Calixarenes as a group of compounds continuously attract the attention of chemists because of their excellent ability to form host–guest complexes. The self-assembled monolayers consisting of calix[n]arene derivatives are applied very often as a sensitive element in sensors designed to detect neutral or ionic analytes [27,28]. Recently, some of these were applied as analytically active elements in sensors dedicated to determining neurotransmitters, such as DA, NEP and EP [21,22,29,30,31,32].

**Figure 1:**
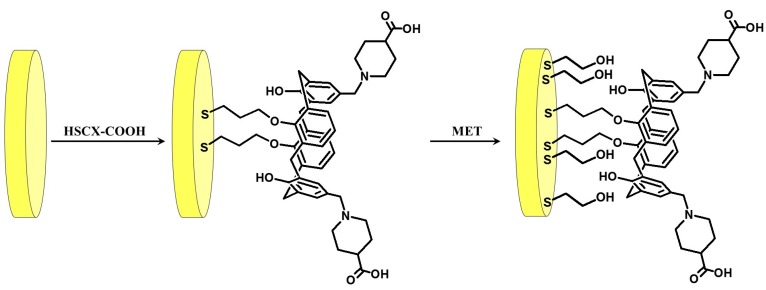
Schematic illustration of the 5,17-bis[*N*-(4-carboxypiperidino)methyl]-25,27-bis(3-mercaptopropoxy)-26,28-dihydroxycalix[4]arene (HSCX-COOH)/ 2-mercaptoethanol (MET) layer preparation.

In the present paper, we have introduced a sensor based on the HSCX-COOH as the host molecule for the detection of DA in the presence of a high concentration of remaining neurotransmitters. Modification of the gold electrode was done according to the scheme presented in Figure 1.

The first step of modification relies on the immobilization of the HSCX-COOH substituted in the bottom rim, with mercaptoalkyl groups added to the surface of the gold electrode through Au–S bonds. In the next step, the pinholes between HSCX-COOH molecules on the Au surface were filled with MET. The presence of the HSCX-COOH/MET layer on the gold electrode surface was confirmed by CV, done according to the procedure described in our previous publication [21]. We have showed that calix[4]arene substituted in the upper rim with two *N*-4-carboxypiperidino groups forming the supramolecular HSCX-COOH–DA complex. The presence of DA as a component of this complex determines it redox activity, and because of that, it could fulfill the role of transducer. Additionally, the presence of the negatively charged carboxylic group should repulse negative charged interfering compounds, such as AA or UA.

**Scheme 1:**
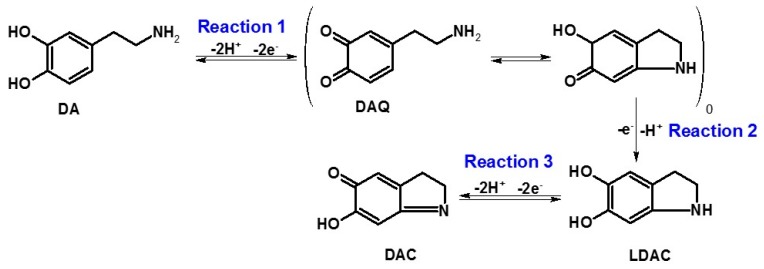
The chemical reaction equations of dopamine oxidation, where DA—dopamine, DAQ—dopamine–*o*–quinone, LDAC—leucodopaminechrome and DAC—dopaminechrome.

DA undergoes a series of reactions classified as an electron transfer−chemical reaction–electron transfer (ECE) mechanism, and its reaction pathway is summarized in Scheme 1. The ECE mechanism describes the course of a reaction sequence in which an irreversible chemical process (reaction 2) takes place between two reversible electrochemical processes (reactions 1 and 3). The first step of the dopamine ECE mechanism involves the facile two-electron oxidation of DA to dopamine-*o*-quinone (DAQ) (reaction 1). The next step involves the irreversible closure of the ring via deprotonation of the amine side chain to leucodopaminechrome (LDAC) (reaction 2). LDAC is then oxidized to dopaminechrome (DAC) (reaction 3). Most researchers have not been interested in reaction 3, but rather, only reaction 1 [33]. 

**Figure 2:**
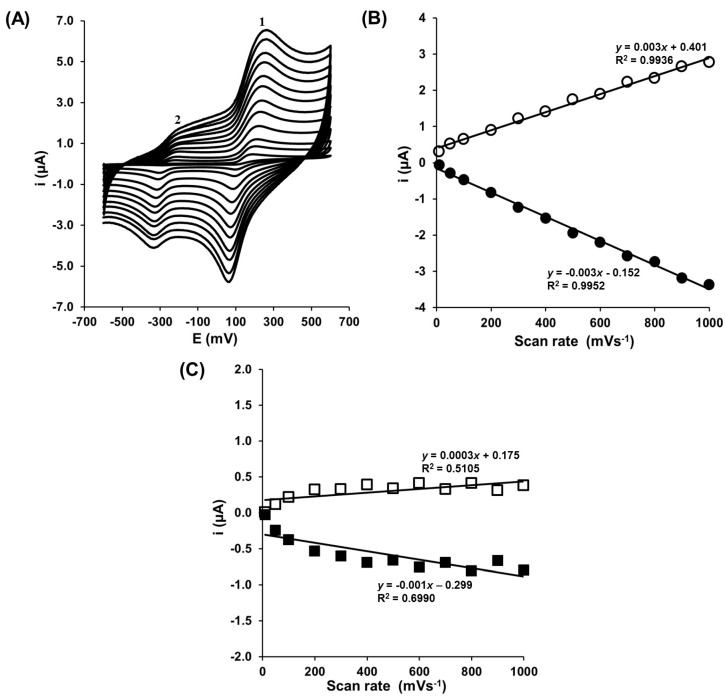
(**A**) Representative cyclic voltammograms recorded for gold electrode modified with HSCX-COOH/MET in the presence of 0.1 mM DA. Scan rate: 10–1000 mV s^−1^; (**B**) Plots of (○) anodic and (●) cathodic current of 0.1 mM DA vs. scan rate for peak 1; (**C**) Plots of (□) anodic and (■) cathodic current of 0.1 mM DA vs. scan rate for peak 2. All measurements were done in phosphate-buffered saline (PBS) pH 7.4.

The presence of HSCX-COOH–DA complex at the surface of the gold electrodes was verified using CV. Figure 2A illustrates the cyclic voltammograms recorded using the gold electrode modified with HSCX-COOH/MET in the presence of 0.1 mM DA in PBS buffer pH 7.4. We have tested the potential range from −600 to +600 mV with a scan rate from 10–1000 mV s^−1^. In these conditions, the two couples of redox peaks were well visible. The first well-defined couple (peak 1) is located in the range of potential from −100 to +600 mV. According to the literature, these redox peaks correlate with an oxidation/reduction of the catechol group of DA/DAQ (reaction 1, Scheme 1) [15,34,35,36,37]. For these two peaks, the relations of cathodic as well as anodic peak current versus the scan rate, are linear (Figure 2B). This indicates that redox reaction, which is going in this range of potential, is not diffusion-dependent and the redox active complex is located at the surface of electrode. The difference between the position of anodic and cathodic peaks current indicates, that the redox reactions in the tested potential range are quasi reversible. The second couple of peaks (peak 2) observed at the potential range from −600 to −250 mV are attributed to the redox reaction of the DA cyclized products (LDAC → DAC) (reaction 3, Scheme 1) [34,36,37,38]. Peak 2 is observed because the reaction 2 rate was fast enough and a sufficient amount of LDAC was generated for the subsequent reaction 3. Part of the generated LDAC was immediately oxidized to DAC, because the rate of reaction 3 is faster than that of reaction 1 [33]. Reaction 3 is less reversible then reaction 1 (Figure 2A). The oxidation peak of the current is very small. Relations of anodic and cathodic peak current versus scan rate are not regular because the electron transfer processes are affected by the kinetics of the chemical step of the cyclization of dopamine (Figure 2C, Scheme 1).

The redox activity of DA depends on the pH conditions as a result of the reaction mechanism, in which protons participate in the electrochemical oxidation/reduction reaction of DA (Scheme 1). The pH effect on the electrochemical responses of DA at Au-HSCX-COOH/MET was investigated using CV (Figure S1A). The redox peak potentials shifted to the negative direction with the increase of pH, indicating that protons were involved in the electrochemical reaction [32]. In the pH range from 2.0 to 7.0, the peak potential shifts linearly toward more negative potentials, with a slope of 48 mV (Figure S1B). This value is close to the Nernstian value of 59 mV at 25 °C, revealing that the number of electrons and protons taking part in the electrode reaction is equal. The differences between these values may be as a result of the ECE mechanism, and that the cyclization process of DA disturbs the reversibility of the oxidation/reduction reaction of dopamine. 

**Figure 3:**
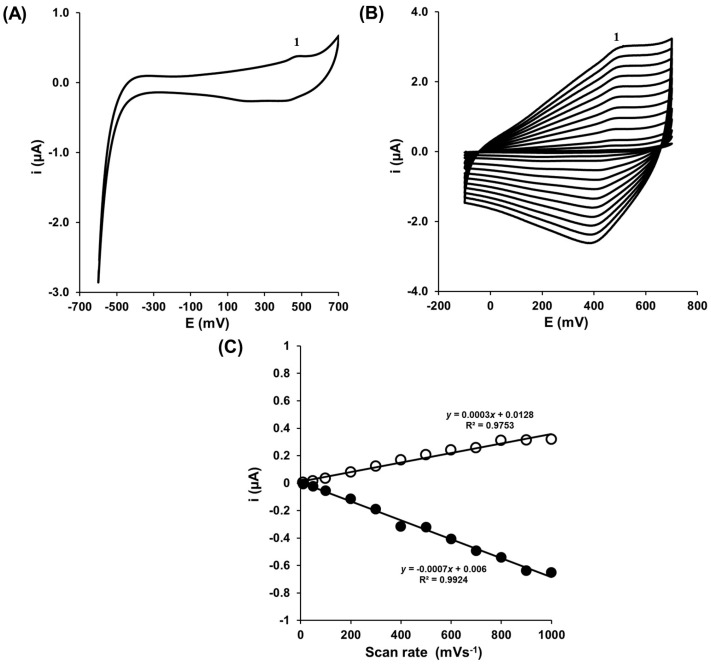
(**A**) Representative cyclic voltammogram recorded for gold electrode modified with HSCX-COOH/MET in the presence of 0.1 mM DA. Scan rate 100 mV s^−1^, potential range from −600 to +600 mV; (**B**) Representative cyclic voltammograms recorded for gold electrode modified with HSCX-COOH/MET in the presence of 0.1 mM DA. Scan rate: 10–1000 mV s^−1^, potential range from −100 to +700 mV; (**C**) Plots of (○) anodic and (●) cathodic current of 0.1 mM DA vs. scan rate for peak 1. All measurements were done in 0.2 M McIlvain buffer pH 2.0.

The presence of DA on the Au-SCX-COOH/MET electrode surface was also confirmed at pH 2.0 in 0.2 M McIlvain buffer. For this purpose, the cyclic voltammograms were recorded for 0.1 mM DA using Au-S-CXCOOH/MET in 0.2 M McIlvain buffer at pH 2.0 in the potential range from −600 to +600 mV (Figure 3A). In this condition only one couple of peaks was observed at the potential range from +380 to +500 mV. This peak was from the oxidation/reduction catechol group of DA/DAQ (reaction 1, Scheme 1) [15,31,32,33,34]. The disappearance of the peak from reaction 3 may be because the rate of reaction 2 in acidic conditions was so slow, that the DAQ was completely consumed in the electron transfer of DAQ → DA before the chemical reaction 2 could go forward [33]. At pH 2.0 the couple of peaks of DA/DAQ were tested for the potential in the range from —100 to +700 mV with scan rate from 10 to 1000 mV s^−1^ (Figure 3B). The linear relationship of the anodic and cathodic peak currents versus the scan rate, shows that the redox processes are not diffusion-dependent and confirms the presence of DA on the gold electrode surface (Figure 3C). 

From an analytical point of view, the reaction 1 (Scheme 1) is much more useful. Therefore, the reaction occurring in the potential range from −100 to +600 mV has been taken into account in the presented sensor destined for the direct electrochemical determination of DA.

**Figure 4:**
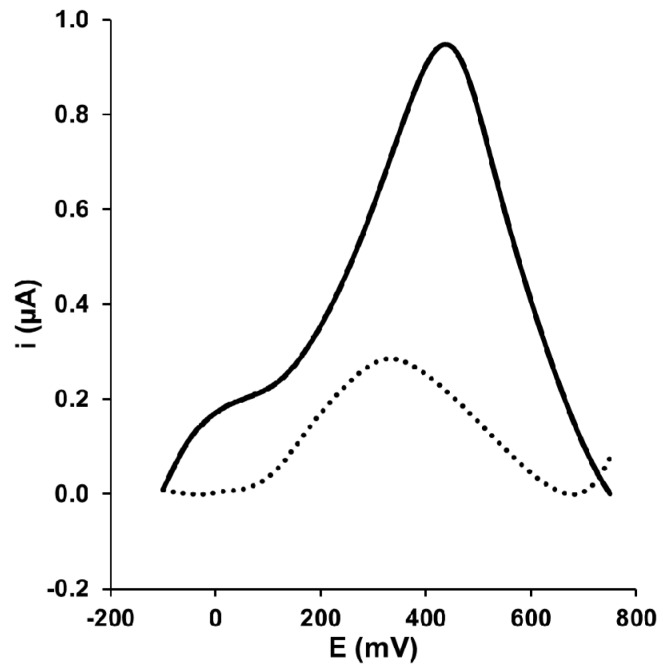
An example of Osteryoung square-wave voltammetry (OSWV) curves recorded with gold electrode HSCX-COOH/MET in the presence of 10 pM DA in 0.2 M McIlvain buffer pH 7.0 (dotted line) and pH 2.0 (solid line). The OSWV was performed with step potential 1 mV, square-wave frequency 25 Hz, and square-wave amplitude 50 mV.

For exploring the DA responses in the selected concentration range, OSWV was applied in order to diminish the capacitive current [21,39,40]. Therefore, the OSWV measurements have been performed in the presence of 10 pM DA, at two different pH 7.0 and 2.0, in the potential range from −100 to +700 mV (Figure 4). The higher current in OSWV and well-defined peak was observed at pH 2.0. According to these results, this condition (pH 2.0) was also kept for exploring DA responses.

### 3.2. Determination of Dopamine in the Presence of Buffer Solution

**Figure 5:**
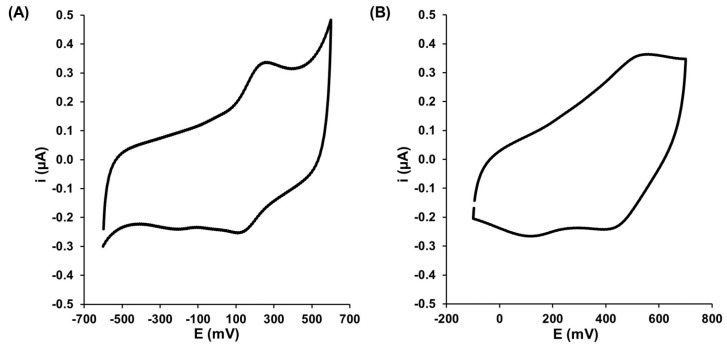
Cyclic voltammogram recorded with electrode Au-SCX-COOH/MET–DA in the blank solution (**A**) PBS buffer pH 7.4 or (**B**) 0.2 M McIlvain pH 2.0.

In the first step, the experiment for the stability confirmation of the HSCX-COOH–DA complex formed at the modified gold electrode surface, was performed. The electrode modified with HSCX-COOH was immersed in 0.1 mM solution of dopamine in the presence of a buffer with pH 7.4 or 2.0 for half hour. Next, the electrode was washed, transferred to the buffer solution without dopamine and tested using CV. Both at pH 7.4 (at +200 mV) and 2.0 (at +500 mV), the visible peak is attributed to the oxidation of DA (Figure 5). This confirms the stability of the HSCX-COOH–DA complex at the gold electrode surface. Similar results were reported by Zheng et al. [22].

The determination of DA using sensors based on the HS-CXCOOH/MET layer deposited on the gold electrode surface was examined with OSWV in two different pH 7.0 and 2.0. 

**Figure 6:**
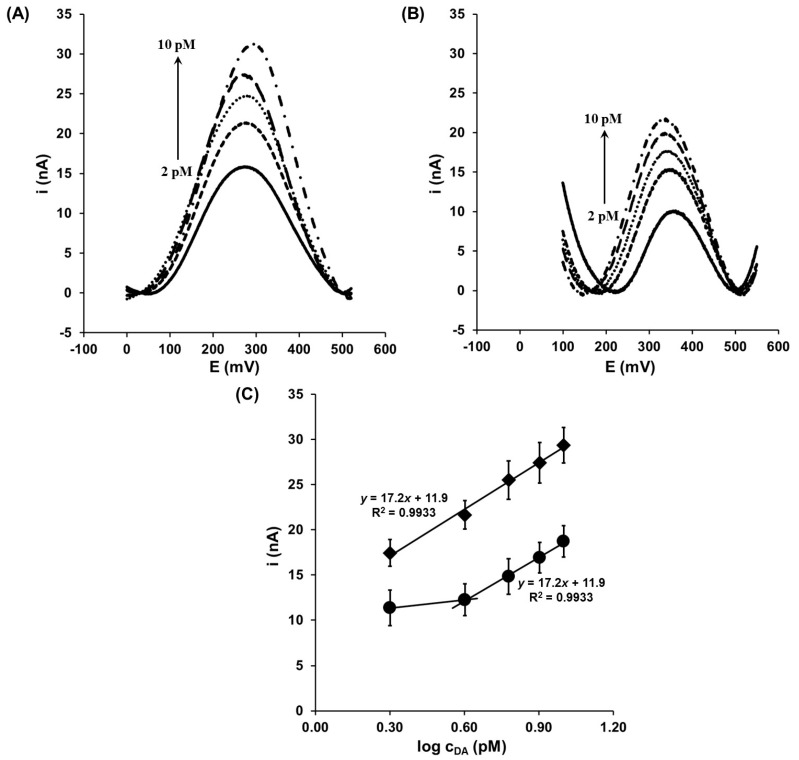
Representative OSWV responses of DA on the HSCX–COOH/MET modified gold electrode (concentration of DA from 2 to 10 pM) in the 0.2 M McIlvain buffer with (**A**) pH 2.0 and (**B**) pH 7.0; (**C**) The intensity of redox DA current vs. the logarithm from the concentration of DA measured with electrodes modified with HSCX–COOH/MET in the 0.2 M McIlvain buffer at (♦) pH 2.0 (*n* = 5, 1.5 < standard deviation (SD) < 2.2) and (●) pH 7.0 (*n* = 5, 1.7 < SD < 2.0). The OSWV was performed with step potential 0.5 mV, square-wave frequency 10 Hz, and square-wave amplitude 25 mV.

With an increasing concentration of dopamine (from 2 to 10 pM), an increase of the DA oxidation/reduction redox current was observed in pH 7.0 as well as pH 2.0 (Figure 6).

The highest concentration of dopamine (10 pM) caused the increase of DA redox current of 29.4 ± 2.0 nA and 18.7 ± 1.7 nA recorded in the buffer solution with pH 2.0 and 7.0, respectively (Figure 6C). This is the consequence of the recognition process between the HSCX-COOH and DA and the formation of the supramolecular complex. This process leads to an increase in the concentration of electroactive DA at the electrode interface. 

The values of the limit of detection (LOD) and the limit of quantification (LOQ) of the proposed sensor were calculated from Equations (1) and (2), respectively:

(1) $$\mathrm{LOD}=\frac{3.3\sigma }{S}$$

(2) $$\mathrm{LOQ}=\frac{10\sigma }{S}$$

where, σ is the standard deviation of the response, and S is the slope of the calibration curve.

In the buffer solution of pH 2.0, the detection limit and limit of quantification were estimated at 1.9 and 7.4 pM. The values of the DA oxidation/reduction current changed linearly within the concentration range from 2 to 10 pM (Figure 6C). In the case of the DA determination in the buffer pH 7.0, the estimated LOD and LOQ were 2.3 and 12.2 pM, respectively. The linear dynamic range in these conditions was in the range of 4 to 10 pM (Figure 6C). 

The optimal pH value for the determination of DA on the Au-SCX–COOH/MET electrode is 2.0, which is selected for evaluating the detection performance of DA in the presence of natural interferences.

### 3.3. Determination of Dopamine in the Presence of Interferents

The presence of electroactive compounds in human plasma, such as -AA and UA, as well as other neurotransmitters, might influence the electrochemical determination of dopamine because of their overlapping oxidation potential [8,41,42]. Therefore, in order to confirm the selectivity of the proposed sensor, the electrochemical determination of DA has been performed in the presence of these potential interferents.

**Figure 7:**
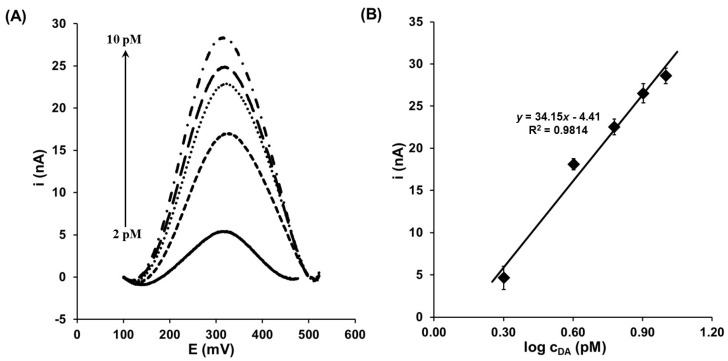
(**A**) Representative OSWVs recorded with gold electrode modified with HSCX-–COOH/MET in the presence of DA at the concentration range from 2 to 10 pM; (**B**) The intensity of redox DA current vs. the logarithm from the concentration of DA measured with electrodes modified with HSCX-–COOH/MET (*n* = 5, 0.7 < SD < 1.4). Measuring conditions: 0.2 M McIlvain buffer pH 2.0 with 1 nM NEP, 1 nM EP, 0.1 mM AA and 0.1 mM UA. The OSWV was performed with step potential 0.5 mV, square-wave frequency 10 Hz, and square-wave amplitude 25 mV.

The concentrations of AA and UA in body fluids are in the 0.1 mM range [8,43,44], while the physiological concentrations of EP and NEP are 0.2 and 1.0 nM, respectively [45]. These potential interferents’ concentrations have been maintained in the buffer destined for dopamine solution preparation. DA electrochemical signals generated in such conditions are presented in Figure 7A.

The obtained results indicated that the presence of the main natural interferents in the sample solution do not have an effect on the dopamine determination with the developed sensor. The linear responses were observed within the concentration range from 2.0 to 10 pM (Figure 7B). The LOD and LOQ, 1.4 and 2.5 pM, respectively, were even better in comparison to these values recorded in the presence of the buffer only.

The calixarenes were already used as an analytical active molecule in electrochemical sensors for the determination of DA. Their analytical parameters are collected in Table 1.

The detection limits of most sensors presented in Table 1 are in the range of µM concentration of DA. There are a few sensors in the literature that allow DA determination in a picomole concentration range. For example, Alatraktchi et al. reported the polyetyleneimine (PEI)-membrane sensor for the determination of DA in the PBS buffer with the LOD at picomolar range (3.1 pM) [46]. This sensor loses sensitivity towards DA in biological material (about three orders of magnitude). In the case of the Au-SCX–COOH/MET sensor, the limit of DA detection achieved by the proposed sensor in pM range in the presence of major interferences, is superior to those already published. It is also worth stressing that the presented sensor allows the selective determination of DA in the presence of other neurotransmitters. The results allow for the improvement of DA monitoring by Au-SCX–COOH/MET electrodes in biological systems, within the 2–10 pM range.

The very high sensitivity of the presented sensor comes from the structure of the supramolecular complex of HSCX-COOH–DA, formed at the gold electrode surface, as a consequence of the intermolecular recognition process. This complex is formed by means of electrostatic interaction between the dissociated carboxylic group of host molecules and protonated amino group of guest molecules. Additionally, the nitrogen from the piperidine ring creates the hydrogen bond with the hydroxyl group of DA. As a result, the DA is located outside of the macrocyclic cavity (Figure S2). Such localization of DA facilitates the run of its redox reaction and, consequently, the strong analytical signal is generated.

## 4. Conclusions

We have introduced a sensor based on gold electrode modified with 5,17-bis[*N*-(4-carboxypiperidino)metyhyl]-25,27,-bis(3-mercaptopropoxy)26,28-dihydroxycalix[4]arene, dedicated to determining DA in the presence of the physiological concentration of AA and UA, as well as EP and NEP. The sensor discussed demonstrated a very good linear dynamic range, from 2.0 to10.0 pM, and a detection limit of 1.4 pM. One of its main advantages is the possibility to determine DA in the presence of other neurotransmitters, AA and UA. This is very important for future sensor applications in physiological samples control. The presented electrochemical sensor is very simple to prepare (which raises the possibility of effortless commercialization), easy to use, and could well be miniaturized. In this sensor, the analytical signal generation is based on the electroactivity of the calix[4]arene–dopamine supramolecular complex. 

## Figures and Tables

**Table 1:** Comparisons of analytical parameters of different electrochemical sensors applied for determination of DA.

Electrode Modification	Measuring Technique	Interferences	Calibration Range (M)	Detection Limit (M)	Reference
Au/Cys/Dopa_RNA aptamer	CA	CH, NEP, EP, L-DOPA, DOPAC, Tyr, HMP	0.1–5.0 × 10^−6^	1.0 × 10^−7^	[15]
Au/Cys/Dopa_RNA aptamer	CV, CA	EP, NEP, L-DOPA, DOPAC, CH, Tyr, HMP	0.1–2.0 × 10^−6^	1.0 × 10^−7^	[16]
Au-Cys/HS-Dopa_RNA aptamer	CA	CT, NEP, L-DOPA	0.1–1.0 × 10^−6^	6.2 × 10^−8^	[1]
Au-PEI membrane	CA	None	3.1 × 10^−12^–1.7 × 10^−2^	3.1 × 10^−12^	[46]
GCE-CACE	CV	AA	0.2 × 10^−6^–1.0 × 10^−3^	3.4 ×10^−6^	[47]
Au/TC8A	DPV	None	1.0 × 10^−6^–1.0 × 10^−3^	5.0 × 10^−7^	[22]
Au/TC6A			3.0 × 10^−6^–1.0 × 10^−3^	8.0 × 10^−7^	
Au/TC4A			5.0 × 10^−6^–1.0 × 10^−3^	1.0 × 10^−6^	
GCE/CUCR-LB	DPV	AA	0.08–6.0 × 10^−6^	2.0 × 10^−8^	[30]
GCE/C4A-LB	DPV	UA, AA	5.0 × 10^−8^–1.0 × 10^−5^	1.5 × 10^−8^	[31]
GCE/RGO-P5A	DPV	UA, AA	1.0–90 × 10^−6^	2.0 × 10^−7^	[32]
Au-SCX-COOH/MET	SWV with addition of redox marker	UA, AA	4.9–12.2 × 10^−12^	4.9 × 10^−12^	[21]
Au-CX-COOH/MET	SWV	NEP, EP, UA, AA	2.0–10.0 × 10^−12^	1.4 × 10^−12^	This work

Au—gold electrode; Cys—cysteamine; Dopa_RNA aptamer—the RNA aptamer for dopamine; CV—cyclic voltammetry; CA—chronoamprometry; CH—catechol; NEP—norepinephrine; EP—epinephrine; L-DOPA—3,4-dihydroxy-phenylalanine; DOPAC—3,4-dihydroxyphenylacetic acid; Tyr—tyramine; HMP—methyldopamine; HS- Dopa_RNA aptamer—the 57-mer 5’-thiol-C6-modified RNA aptamer specific for dopamine; PEI membrane—polyetyleneimine membrane; CACE—calix[4]arene crown-4 ether; DPV—differential pulse voltammetry; AA—ascorbic acid; UA—uric acid; TC8A—thiolated calix[8]arene; TC6A—thiolated calix[6]arene; TC4A—thiolated calix[4]arene; GCE—glassy carbon electrode; CUCR—C-Undecylcalix[4]resorcinarene; LB—Langmuir-Blodgett; C4A—calix[4]resorcinarene; RGO—reduced graphen oxide; P5A—pillar[5]arene; MET—2-mercaptoethanol; HSCX-COOH—5,17-Bis[*N*-(4-carboxypiperidino)methyl]-25,27-bis(3-mercaptopropoxy)-26,28-dihydroxycalix[4]arene; SWV—square-wave voltammetry.

## Supplementary Materials

The following data are available online at http://www.mdpi.com/1424-8220/17/6/1368/s1, Figure S1: (A) Representative cyclic voltammograms of 10 pM DA at Au-S-CXCOOH/MET in 0.2 M McIlvain buffer pH 2.0 (red line) and pH 7.0 (blue line). Scan rate: 100 mV s^−1^, (B) The linear relationship between the dopamine oxidation potential and pH; Figure S2: Scheme of supramolecular complex Au-SCX-COOH/MET–dopamine.

## References

[1] Álvarez-Martos I., Ferapontova E.E. (2016). Electrochemical label-free aptasensor for specific analysis of dopamine in serum in the presence of structurally related neurotransmitters. Anal. Chem..

[2] Grace A. (1991). Phasic versus tonic dopamine release and the modulation of dopamine system responsivity: A hypothesis for the etiology of schizophrenia. Neuroscience.

[3] Obeso J., Rodriguez-Oroz M.C., Geotz C.G., Marin C., Kordower J.H., Rodriguez M., Hirsch E.C., Farrer M., Schapira A.H., Haliday G. (2010). Missing pieces in the Parkinson's disease puzzle. Nat. Med..

[4] Tong J., Hornykiewicz O., Kish S.J. (2006). Inverse relationship between brain noradrenaline level and dopamine loss in Parkinson Disease—A possible neuroprotective role for noradrenaline. Arch. Neurol..

[5] Panholzer T.J., Beyer J., Lichtwald K. (1999). Coupled-column liquid chromatographic analysis of catecholamines, serotonin, and metabolites in human urine. Clin. Chem..

[6] Chauveau J., Fert V., Morel A.M., Delaage M.A. (1991). Rapid and specific enzyme immunoassay of serotonin. Clin. Chem..

[7] Middelkoop C.M., Dekker G.A., Kraayenbrink A.A., Popp-Snijders C. (1993). Platelet-poor plasma serotonin in normal and preeclamptic pregnancy. Clin. Chem..

[8] Jackowska K., Krysinski P. (2013). New trends in the electrochemical sensing of dopamine. Anal. Bioanal. Chem..

[9] Kissinger P.T., Hart J.B., Adams R.N. (1973). Voltammetry in brain tissue—A new neurophysiological measurement. Brain Res..

[10] Rozniecka E., Jonsson-Niedziołka M., Celebanska A., Niedziolka-Jonsson J., Opallo M. (2014). Selective electrochemical detection of dopamine in a microfluidic channel on carbon nanoparticulate electrodes. Analyst.

[11] Xiang L., Lin Y., Yu P., Su L., Mao L. (2007). Laccase-catalyzed oxidation and intramolecular cyclization of dopamine: A new method for selective determination of dopamine with laccase/carbon nanotube-based electrochemical biosensors. Electrochim. Acta.

[12] Du J., Yue R., Ren F., Yao Z., Jiang F., Yang P., Du Y. (2014). Novel graphene flowers modified carbon fibers for simultaneous determination of ascorbic acid, dopamine and uric acid. Biosens. Bioelectron..

[13] Viry L., Derré A., Poulin P., Kuhn A. (2010). Discrimination of dopamine and ascorbic acid using carbon nanotube fiber microelectrodes. Phys. Chem. Chem. Phys..

[14] Ferapontova E.E., Castillo J., Gorton L. (2006). Bioelectrocatalytic properties of lignin peroxidase from Phanerochaete chrysosporium in reactions with phenols, catechols and lignin-model compounds. Biochim. Biophys. Acta.

[15] Farjami E., Campos R., Nielsen J.S., Gothelf K.V., Kjems J., Ferapontova E.E. (2013). RNA aptamer-based electrochemical biosensor for selective and label-free analysis of dopamine. Anal. Chem..

[16] Álvarez-Martos I., Camposa R., Ferapontova E.E. (2015). Surface state of the dopamine RNA aptamer affects specific recognition and binding of dopamine by the aptamer-modified electrodes. Analyst.

[17] Neri P., Sessler J.L., Wang M.-X. (2016). Calixarenes and Beyond.

[18] Nimse S.B., Kim T. (2013). Biological applications of functionalized calixarenes. Chem. Soc. Rev..

[19] Arora V., Chawla H.M., Singh S.P. (2007). Calixarenes as sensor materials for recognition and separation of metal ions. ARKIVOC.

[20] Ludwig R., Dzung N.T.K. (2002). Calixarene-based molecules for cation recognition. Sensors.

[21] Kurzątkowska K., Sayin S., Yilmaz M., Radecka H., Radecki J. (2015). Calix[4]arene derivatives as dopamine hosts in electrochemical sensors. Sens. Actuators.

[22] Zheng G., Chen M., Liu X., Zhou J., Xie J., Diao G. (2014). Self-assembled thiolated calix[n]arene (*n* = 4, 6, 8) films on gold electrodes and application for electrochemical determination dopamine. Electrochim. Acta.

[23] Wang F., Wu Y., Lu K., Ye B. (2013). A simple but highly sensitive and selective calixarene-based voltammetric sensor for serotonin. Electrochim. Acta.

[24] Umezawa Y., Aoki H. (2004). Ion channel sensors based on artificial receptors. Anal. Chem..

[25] Radecka H., Szymańska I., Pietraszkiewicz M., Pietraszkiewicz O., Aoki H., Umezawa Y. (2005). Intramolecular ion-channel sensors using gold electrodes immobilized with macrocyclic polyamines for voltammetric detection of adenine nucleotides. Anal. Chem..

[26] Radecki J., Szymańska I., Bulgariu L., Pietraszkiewicz M. (2006). Covalent and embedment immobilization of macrocyclic polyamines on gold electrodes and their voltammetric responses towards ethene dicarboxylic acids. Electrochim. Acta.

[27] Kim H.J., Lee M.H., Mutihac L., Vicens J., Kim J.S. (2012). Host-guest sensing by calixarenes on the surfaces. Chem. Soc. Rev..

[28] Poduval R., Kurzątkowska K., Stobiecka M., Dehaen W.F.A., Dehaen W., Radecka H., Radecki J. (2010). Systematic study of interaction of the neutral form of anilines with undecylcalix[4]resorcinarene derivatives by means of potentiometry. Supramol. Chem..

[29] Doyle R., Breslin C.B., Rooney A.D. (2009). A Simple But highly selective electrochemical sensor for dopamine. Chem. Biochem. Eng. Q..

[30] Wang F., Chia C.L., Yua B., Ye B. (2015). Simultaneous voltammetric determination of dopamine and uric acid based on Langmuir-Blodgett fil of calixarene modified glassy carbon electrode. Sens. Actuators.

[31] Wang F., Wu Y., Ma H., Li B., Ye B. (2016). A simple but highly sensitive and selective electrochemical sensor for determination of dopamine based on Langmuir-Blodgett film of calix[4]resorcinarene. J. Electrochem. Soc..

[32] Liu X., Wang W., Li X., Li C., Qin L., Sun J., Kang S.-Z. (2016). Preparation of per-hydroxylated pillar[5]arene decorated graphene and its electrochemical behavior. Electrochim. Acta.

[33] Muguruma H., Inoue Y., Inoue H., Ohsawa T. (2016). Electrochemical study of dopamine at electrode fabricated by cellulose-assisted aqueous dispersion of long-length carbon nanotube. J. Phys. Chem..

[34] Hawley M.D., Tatawawadi S.V., Piekarski S., Adams R.N. (1967). Electrochemical studies of the oxidation pathways of catecholamine. J. Am. Chem. Soc..

[35] Baur J.E., Kristensen E.W., May L.J.M., Wiedemann D.J., Wigthman R.M. (1988). Fast-scan voltammetry of biogenic amines. Anal. Chem..

[36] Gu X., Jiang G., Jiang G., Chen T., Zhan W., Li X., Wu S., Tian S. (2015). Detection of dopamine on a mercapto-terminated hexanuclear Fe(III) cluster modified gold electrode. Talanta.

[37] Ghica M.E., Brett C.M.A. (2013). Simple and efficient epinephrine sensor based on carbon nanotube modified carbon film electrodes. Anal. Lett..

[38] Hu M., Fritsch I. (2016). Application of electrochemical redox cycling: Toward differentiation of dopamine and norepinephrine. Anal. Chem..

[39] Jagiło A., Grabowska I., Radecka H., Sulima M., Marszałek I., Wysłouch-Cieszyńska A., Dehaen W., Radecki J. (2013). Redox active dipyrromethene—Cu(II) monolayer for oriented immobilization of His-tagged RAGE domains—The base of electrochemical biosensor for determination of Aβ_16–23_. Electroanalysis.

[40] Malecka K., Grabowska I., Radecki J., Stachyra A., Góra-Sochacka A., Sirko A., Radecka H. (2012). Voltammetric detection of a specific DNA Ssequence of Avian Influenza Virus H5N1 using HS-ssDNA probe deposited onto gold electrode. Electroanalysis.

[41] Patel A.N., Tan S.-Y., Miller T.S., Macpherson J.V., Unwin P.R. (2013). Comparison and reappraisal of carbon electrodes for the voltammetric detection of dopamine. Anal. Chem..

[42] Šnejdárková M., Poturnayová A., Rybár P., Lhoták P., Himl M., Flídrová K., Hianki T. (2010). High sensitive calixarene-based sensor for detection of dopamine by electrochemical and acoustic methods. Bioelectrochemistry.

[43] Xu T.-Q., Zhang Q.-L., Zheng J.-N., Lv Z.-Y., Wei J., Wang A.-J., Feng J.-J. (2014). Simultaneous determination of dopamine and uric acid in the presence of ascorbic acid using Pt nanoparticles supported on reduced graphene oxide. Electrochem. Acta.

[44] Tan S., Radi S.R., Gaudier F., Evans R.A., Rivera A., Kirk K.A., Parks D.A. (1993). Physiologic levels of uric acid inhibit xanthine oxidase in human plasma. Pediatr. Res..

[45] Wittstein I.S., Thiemann D.R., Lima J.A.C., Baughman K.L., Schulman S.P., Gerstenblith G., Wu K.C., Rade J.J., Bivalacqua T.J., Champion H.C. (2005). Neurohumoral features of myocardial stunning due to sudden emotional stress. N. Engl. J. Med..

[46] AlZahra’a Alatraktchi F., Bakmand T., Dimaki M., Svendsen W.E. (2014). Novel membrane-based electrochemical sensor for real-time bio-applications. Sensors.

[47] Lai G.-S., Zhang H.-L., Jina C.-M. (2007). Electrocatalysis and voltammetric determination of dopamine at a calix[4]arene crown-4 ether modified glassy carbon electrode. Electroanalysis.

