# Supplementary material for: Gold Electrodes Modified with Calix[4]arene for Electrochemical Determination of Dopamine in the Presence of Selected Neurotransmitters
Source: Sensors (Basel). 2017 Jun 13;17(6):1368. doi: 10.3390/s17061368 (PMC5492125; doi:10.3390/s17061368)
Supplement: Supplementary file 1 [file sensors-17-01368-s001.pdf]

sensors

Supplementary Materials

Gold Electrodes Modified with Calix[4]arene for Electrochemical Determination of Dopamine in the Presence of Selected Neurotransmitters

Katarzyna Kurzątkowska [1], Serkan Sayin [2], Mustafa Yilmaz [3], Hanna Radecka [1] and Jerzy Radecki [1,*]

[1] Department of Biosensors, Institute of Animal Reproduction and Food Research Polish Academy of Science, Tuwima 10 Street, 10-748 Olsztyn, Poland
[2] Department of Environmental Engineering, Faculty of Engineering, Giresun University, Giresun-28200, Turkey
[3] Department of Chemistry, Selcuk University, Konya, Turkey
* Correspondence: j.radecki@pan.olsztyn.pl; Tel.: +48-89-523-46-12

CONTENTS

Figure 1S. (**A**) Representative cyclic voltammograms of 10 pM DA at Au-SCX-COOH/MET in 0.2 M McIlvain buffer pH 2.0 (red line) and pH 7.0 (blue line). Scan rate: 100 mV s^{-1}. (**B**) The linear relationship between the dopamine oxidation potential and pH.

Figure 2S. Scheme of supramolecular complex Au-SCX-COOH/MET–dopamine.

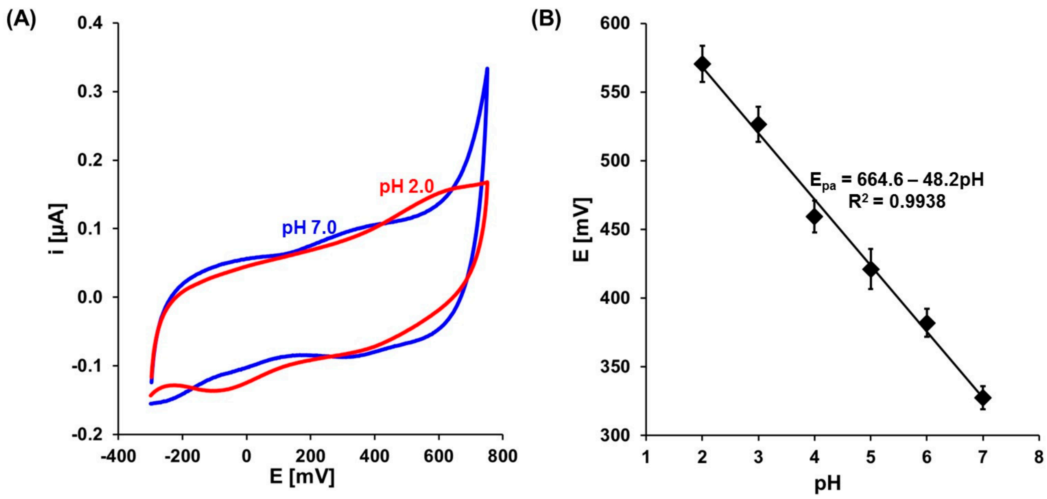

Figure 1S. (**A**) Representative cyclic voltammograms of 10 pM DA at Au-SCX-COOH/MET in 0.2 M McIlvain buffer pH 2.0 (red line) and pH 7.0 (blue line). Scan rate: 100 mV s^{-1}. (**B**) The linear relationship between the dopamine oxidation potential and pH.

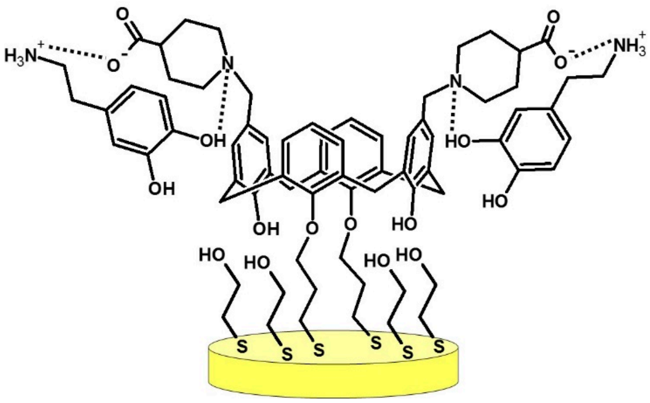

Figure 2S. Scheme of supramolecular complex Au-SCX-COOH/MET–dopamine.

© 2017 by the authors. Licensee MDPI, Basel, Switzerland. This article is an open access article distributed under the terms and conditions of the Creative Commons Attribution (CC BY) license (http://creativecommons.org/licenses/by/4.0/).
